# Preparation of Cobalt Oxide–Reduced Graphitic Oxide Supercapacitor Electrode by Photothermal Processing

**DOI:** 10.3390/nano11030717

**Published:** 2021-03-12

**Authors:** Madhu Gaire, Najma Khatoon, Douglas Chrisey

**Affiliations:** Department of Physics and Engineering Physics, School of Science and Engineering, Tulane University, New Orleans, LA 70118, USA; nkhatoon@tulane.edu (N.K.); dchrisey@tulane.edu (D.C.)

**Keywords:** photonic synthesis, spray coating, cobalt oxides, pseudocapacitor, synergistic effect

## Abstract

We report a photonic technique to instantaneously synthesize cobalt oxide reduced graphitic oxide (CoO_x_-rGO) supercapacitor electrodes. The electrode processing is achieved through rapidly heating the precursor material by irradiation of high-energy pulsed mostly visible light from a xenon lamp. Due to the short duration of the light pulse, we prepared the electrodes at room temperature instantaneously (ms), thus eliminating the several hours of processing times of the conventional techniques. The as-prepared electrodes exhibited a highly porous morphology, allowing for enhanced ionic transport during electrochemical interactions. The electrochemical properties of the CoO_x_-rGO electrodes were studied in 1 M KOH aqueous electrolyte. The non-rectangular cyclic voltammetry (CV) curves with characteristic redox peaks indicated the pseudocapacitive charge storage mechanism of the electrodes. From the discharge curves at 0.4 mA/cm^2^ and 1.6 A/g constant current densities, the electrode showed areal specific capacitance of 17 mF/cm^2^ and specific capacitance of 69 F/g, respectively. Cyclic stability was tested by performing 30,000 galvanostatic charge–discharge (GCD) cycles and the electrode exhibited 65% capacitance retention, showing its excellent electrochemical performance and ultra-long cycle life. The excellent electrochemical electrode properties are attributed to the unique processing technique, optimum processing parameters, improved conductivity due to the presence of rGO, and highly porous morphology which offers a high specific surface area. The novel photonic processing we report allows for high-temperature heating of the precursor films achieved via non-radiative recombination of photogenerated electron holes pairs during irradiation. Such extremely quick (ms) heating followed by instantaneous cooling results in the formation of a dense and robust bottom layer of the electrode, resulting in a long cycle life.

## 1. Introduction

Supercapacitors (SCs) have attracted significant interest as efficient energy storage devices because of their high-power density, fast charge–discharge ability, and long cycle life [1,2,3,4]. Due to these attributes, supercapacitors have been used in portable electronics, electric vehicles, and smart toys, etc. However, their low energy density compared to batteries is the main issue that needs to be resolved. To this end, the researchers have been working on developing novel electrode materials that can provide high specific surface area and high electrical conductivity. Based on the charge storage mechanism, the supercapacitors can be further categorized as electric double-layer capacitors (EDLCs) with carbon-based electrodes, and pseudocapacitors with metal oxides and conducting polymer electrodes. In EDLCs, the charge storage on the electrode’s surface is achieved through physical adsorption, whereas, in pseudocapacitors, the charge storage is achieved via Faradaic redox reactions between electrolyte ions and the active materials at the electrode-electrolyte interface [5,6,7]. Due to these fast and reversible redox reactions, metal oxides provide higher energy density than EDLCs based on carbonaceous materials [1,8,9].

Among metal oxides, cobalt oxide (CoO_x_) is widely studied as a supercapacitor electrode due to its low cost, environmental benignity, and good redox properties [4]. However, pure metal oxides, such as cobalt oxides have low conductivity and shorter cycle life than carbon materials, limiting their practical applications. Furthermore, during the charge–discharge processes, pure metal oxide electrode goes through continuous volume changes, resulting in poor structural integrity. To resolve these issues, we report the incorporation of cobalt oxides with carbon materials to fabricate composite electrodes with high capacitance as well as long cycle life, eliminating the shortcomings of pure metal oxides. This is possible because carbon materials offer high electronic conductivity, enhanced structural and thermal stability, and high accessible surface area, resulting in improved capacitance and cyclic life [6]. Furthermore, the synergistic effect between carbon materials and metal oxide ensures that the composite electrodes have better electrochemical performance than either one of them individually could have.

Generally, the electrochemical performance of an electrode depends significantly on its structure and morphology as well as the choice of substrate and the growth mechanism of the active materials. Therefore, the fabrication of electrodes with novel surface structures is of crucial importance. In this regard, we report the fabrication of nanostructured and highly porous cobalt oxides reduced graphitic oxides (CoOx-rGO) electrodes with high specific surface area, offering more active sites for electrode/electrolyte interactions. Additionally, such porous morphology of the electrodes not only offers increased utilization of the active materials but also accommodates the volume changes during electrochemical processes, which are crucial for long cyclic life. By preparing the binder-free nanoparticles on a highly conductive substrate, the electronic conductivity of the electrode could be enhanced and mechanical adhesion between the active material and the substrate could be reinforced. Additionally, by preparing CoO_x_-rGO composite, the overall performance of the electrode can be further improved due to excellent electrical and mechanical properties of rGO, such as high surface area, high electrical conductivity, and good electrochemical stability [6,10]. More importantly, the electrodes can utilize the pseudocapacitance from metal oxides as well as double-layer capacitance from rGO, leading to higher specific capacitance.

Various methods [1,11,12,13,14,15] have been reported for the preparation of cobalt oxide electrodes taking several hours of processing time. Herein, we report a novel technique based on the photothermal treatment of the precursor material that can be accomplished instantaneously, eliminating the long processing times of already reported conventional techniques. Additionally, unlike already reported techniques, this novel photothermal technique is low-cost, highly scalable to meet industrial demands, suitable for inexpensive substrates, has a small footprint, and offers ultrahigh speed (ms) and room temperature processing. To the best of our knowledge, this is the shortest processing time reported for the fabrication of CoO_x_-rGO supercapacitor electrodes.

The applicability of the as-prepared electrode was evaluated by using cyclic voltammetry (CV), galvanostatic charge–discharge (GCD) test and electrochemical impedance spectroscopy (EIS) in 1 M KOH aqueous electrolyte. Following the measurement, the electrode showed an initial specific capacitance of 17 mF/cm^2^ at 0.4 mA/cm^2^ current density. The electrochemical stability and cycle life were studied by performing 30,000 GCD cycles at 0.4 mA/cm^2^ current density, and the electrode maintained 65% capacitance retention, indicating its excellent capacitive performance and long cycle life.

## 2. Materials and Methods

### 2.1. Spray-Coating Thin Film Using an Air-Spray

**Figure 1:**
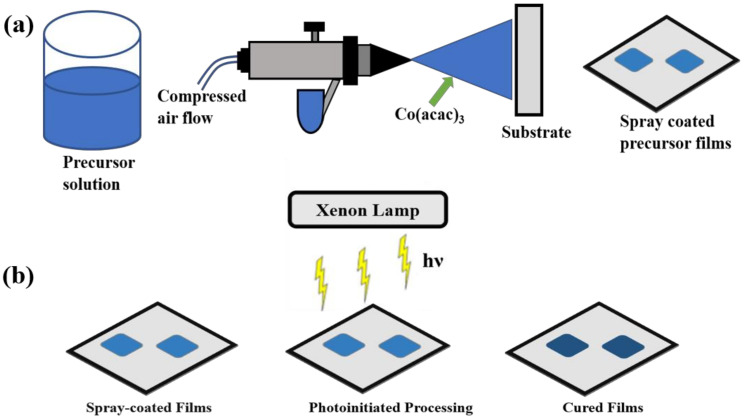
Schematic diagram of thin film (**a**) spray-coating technique and (**b**) curing using photoinitiated processing method.

The precursor solution was prepared by dissolving 0.5 g organometallic precursor powder Co (III) acetylacetonate (98.5%, Sigma-Aldrich, St. Louis, MO, USA) in 20 mL acetone (99.5%, Sigma-Aldrich, St. Louis, MO, USA) and placed in an ultrasonication system for 20 min. Thin-film deposition was accomplished by spray-coating, which we reported in our past work [16,17], the precursor solution on 1 cm × 1 cm Pt-coated silicon wafer (University Wafer, Boston, MA, USA) using an air-spray (Paasche Airbrush, Chicago, IL, USA) in ambient conditions. To ensure uniformity of the films, parameters such as the gas flow, precursor solution concentration, distance between the tip of the spray nozzle and the substrate, etc. were kept constant. The thin film deposition was accomplished in several seconds and adherent powder precursor films were prepared. Figure 1a shows the schematic representation of thin film deposition using an air-spray.

### 2.2. Preparation of CoO_x_-rGO Electrodes

A novel photonic curing technique (PulseForge 1300, NovaCentrix Corp., Austin, TX, USA) was used to process the as-sprayed films to prepare thin-film electrodes almost instantaneously. Photonic curing involved continuous irradiation of the as-sprayed precursor electrodes with high energy pulsed light from a xenon lamp. The energy density of the pulsed light was measured with in situ bolometer before starting the irradiation. Processing parameters, such as the number of pulses, voltage, pulse length, number of micro pulses, etc. are shown in Table S1 (ESI), and could be easily controlled by a computer. We processed electrodes with 2, 25, and 100 pulses while keeping other parameters the same for all the electrodes. Since cobalt acetylacetonate precursor absorbs the visible light, the photonic curing by irradiating the precursor material with light from a xenon lamp with an output spectrum between 220 and 1000 nm is a viable technique to prepare the electrodes. Due to the extremely short pulse duration (ms), the processing could be achieved instantaneously, leaving the substrate underneath intact. After the photonic processing, initially non-conductive and adherent powder precursor films were transformed into dense and conductive CoO_x_-rGO thin-film electrodes. Figure 1b schematically represents the photonic processing mechanism of the electrodes. The mass loading of the electrode after processing was calculated to be 0.25 mg.

### 2.3. Photothermal Processing and Nanostructure Growth Mechanism

While conventional thermal processing methods involve exposing thin film samples to high temperatures for several hours thus limiting the processing speed and the choice of substrates, pulsed photonic curing offers ultra-high-speed (ms) and is suitable for the use of low cost and flexible substrates thanks to the short duration and short penetration depth of highly energetic pulsed light from a xenon lamp which produces light with a broad spectrum of 200–1200 nm. Additionally, unlike laser-based surface annealing with extremely (ns) short exposure, a small area (~1 cm^2^), and fixed wavelength, pulsed photonic processing offers (a) a large exposure area (~100 cm^2^), (b) a wide wavelength range, (c) roll-to-roll processing capability with flexible substrates, and (d) ability to heat the precursor films to ~1000 °C over a short time (ms) without damaging the substrate. Since the photonic curing technique is purely based on the absorption of the light by precursor materials, to achieve high-temperature processing in a short period of time, the precursor materials must have high optical absorption characteristics, high thermal conductivity (>50 Wm^−1^K^−1^), and specific heat capacity (>200 J kg^−1^K^−1^). In this regard, metal acetylacetonate precursors, such as cobalt acetylacetonate, manganese acetylacetonate, etc. are strong absorbers of high-intensity visible light, making photonic processing technique a novel approach for instantaneous fabrication of thin-film electrodes for pseudocapacitors and likely other applications.

The photonic curing involves (i) evaporation of residual solvent, (ii) decomposition of the precursor, (iii) agglomeration and sintering of the precursor particles to create a conductive thin film electrode. During the photonic curing process, the precursor material absorbs the high-intensity photons from different mostly visible wavelengths and converts them into lattice phonons and thermal energy within the ms time range. As a result, rapid photothermal heating takes place throughout precursor material, resulting in high temperatures (~1500 °C) creating an extreme thermal gradient between the thin film sample and the substrate. After the pulse train, the thermal energy in the films will dissipate quickly (ns) in three different ways [18]: (a) radiation relaxation where energy re-emits in the form of radiation, (b) charge separation, where energy is consumed by the formation of the electron–hole pairs, and (c) non-radiation relaxation where energy is conducted away by atomic lattice vibrations known as phonons [19]. Among these, during photothermal treatment of the precursor, the heat energy can be dissipated quickly throughout the bulk of the film through non-radiation relaxation process, resulting in higher temperature needed for sintering of the precursor particles.

The first few micro pulses of the irradiation evaporate the residual solvent in the films, decreasing the separation between the particles, which leads to the agglomeration of the particles and spreading of thermal energy throughout the bulk of the precursor. With subsequent micro pulses, the precursor material absorbs sufficient energy, resulting in thermal atomic motion of the precursor particles and overcoming the energy barrier to initiate organic decomposition and local crystallization. This is followed by the formation and activation of the nanoparticles, nucleation and growth of nanoparticles, and eventually the formation of the sintered and nanostructured electrode. As the particles reach the nanoscale dimension, the heat generated from photothermal treatment will be trapped in the individual nanostructures [20]. This means that the non-radiative recombination of photogenerated electron and holes in as-formed nanostructures results in inefficient heat dissipation to neighboring nanostructures, resulting in high local temperature, and providing sufficient thermodynamic driving force to improve the crystalline quality of the as-processed samples.

During the pulse light irradiation, the heat absorbed by the sample is strongly dependent on its absorption coefficient, thickness, and the reflection from its surface. The Beer-Lambert law gives the relationship between the total absorption of the light, absorptivity (*α*) and thickness (*x*) of the precursor films. During the pulsed light irradiation, for a fixed area, A, the amount of heat absorbed by the sample can be calculated by the following equation [21]:

$$Q=A\int \int I_{0}\left(\lambda \right)e^{-\alpha x}d\lambda dx$$

where A, $I_{0}$, α, and x are the illuminated area, the intensity of incident radiation, absorptivity, and thickness of the films, respectively.

### 2.4. Characterization

The morphology of the as-prepared electrodes was investigated by using a field-emission scanning electron microscopy (FE-SEM) (Hitachi S-4800, Hitachi Corp., Tokyo, Japan). To analyze the crystalline structure and the composition of the electrodes, DXR Raman Microscope (Thermo Scientific, Waltham, MA, USA) with a 532 nm laser with 5 mW power was used to record the Raman spectra. Energy-dispersive X-ray spectroscopy (EDX) (Hitachi S-3400, Hitachi Corp., Tokyo, Japan) was performed to verify the elemental composition of the electrodes.

### 2.5. Electrochemical Measurements

**Figure 2:**
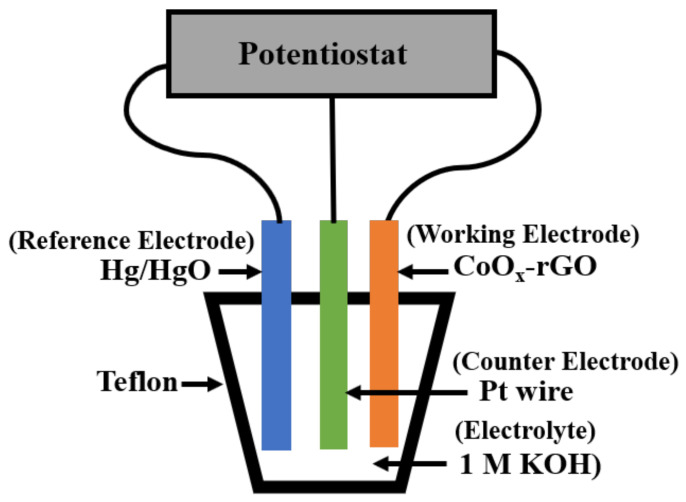
Schematic representation of the three-electrode measurement arrangement.

To perform the electrochemical measurements, an electrochemical workstation (Metrohm Autolab B.V., Utrecht, The Netherlands) was used with a three-electrode configuration, where the as-prepared CoO_x_-rGO, Hg/HgO and Pt wire were working, reference and counter electrodes, respectively. The electrochemical measurements included the cyclic voltammetry (CV), galvanostatic charge–discharge test (GCD) and electrochemical impedance spectroscopy (EIS). CV measurements were performed at different scan rates from 10 to 100 mV/s in the potential window of 0–0.6 V. The GCD tests were carried out in the same potential window and at various applied current densities. The EIS measurements were recorded in a frequency range of 100 kHz to 10 mHz with an ac perturbation signal of 10 mV. All the electrochemical measurements were carried out in 1 M KOH aqueous electrolyte in ambient conditions at room temperature (23 °C). The electrochemical measurements setup was constructed using the PTFE (Teflon) rod (McMaster-Carr, Elmhurst, IL, USA), and it is schematically shown in Figure 2.

## 3. Results and Discussion

**Figure 3:**
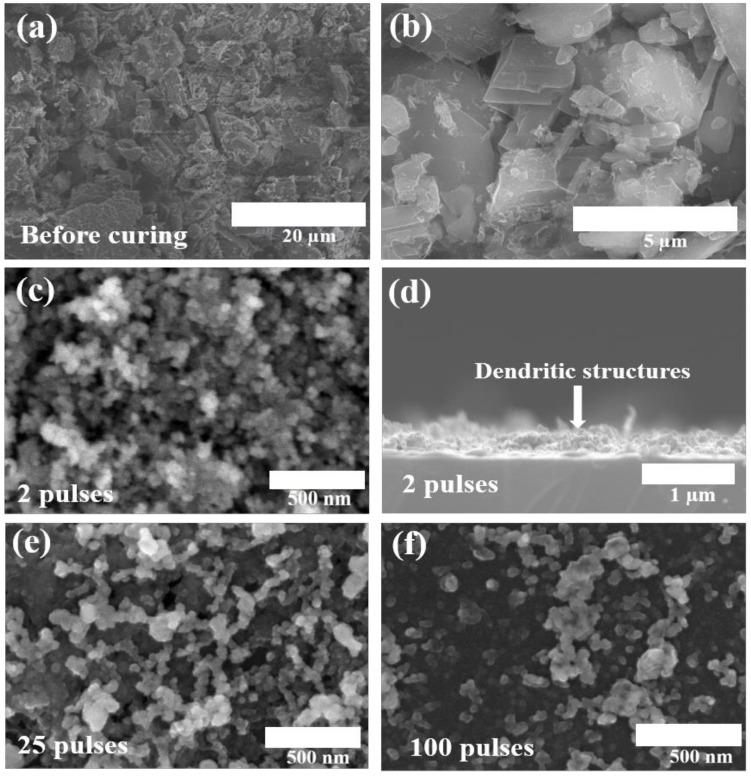
SEM images of precursor thin film sample before curing (**a**,**b**). SEM surface and cross-sectional morphology, respectively, of 2 pulses cured electrode (**c**,**d**), and surface morphology of 25 and 100 pulses cured electrodes (**e**,**f**).

The electrochemical performance of the pseudocapacitor depends on its structure and morphology as the capacitance originates from the surface-based interaction between the active material and the electrolyte. Therefore, nanostructure and porosity play a crucial role in the electrode’s electrochemical performance [22]. For the high performance of the electrodes, it is required that the surface be porous, preventing the aggregation of the active material. The morphologies of the as-prepared electrodes before and after processing are shown in Figure 3. The spray-coated electrodes (before curing) have dense morphology and contained particles of a size around 2 µm, as shown in Figure 3a,b. During the photothermal treatment of the precursor electrodes, because of the unique processing mechanism of the photonic curing system, the active material absorbs enough energy to go through electrochemical decomposition and form highly porous and uniformly grown nanostructures with an average particle size of 50 nm. Such structures offer a high specific surface area for electrode/electrolyte interaction, shorten the ion diffusion pathways, accommodate the volume changes during charge/discharge leading to the enhanced structural integrity of the electrode, and allow for the electrolyte ions to penetrate deep into the active material. Figure 3c,e,f and Figure S1 (ESI) show the surface morphology of the as-prepared electrodes cured with 2, 25, and 100 pulses, respectively. Figure 3d shows the cross-sectional morphology of the two pulses cured electrode, clearly showing a top dendritic layer and a bottom dense layer. While the top porous layer allows for the enhanced diffusion of ions and offers a high surface area for electrolyte/electrode interactions, the bottom layer keeps the structure of the electrode robust during charge–discharge cycles.

**Figure 4:**
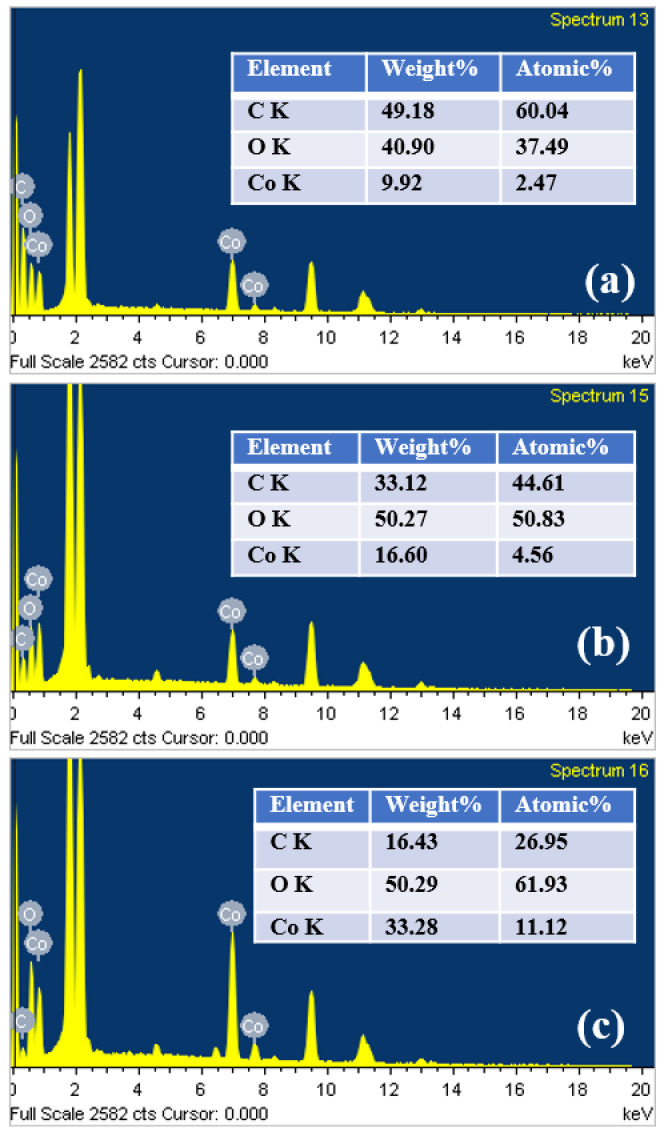
EDX spectra of the electrodes cured with (**a**) 2 pulses, (**b**) 25 pulses and (**c**) 100 pulses, respectively.

To investigate the elemental composition of the as-prepared electrodes, EDX spectra were recorded, as shown in Figure 4. It can be clearly observed that all the electrodes consist of C, O and Co elements with different atomic%. The intense peaks in the 2 keV range are associated with the substrate. Remarkably, as the number of pulses increased, the electrodes showed a significant reduction in the weight percentage of carbon. This is possibly due to the thermal evaporation of the carbon component in the films because of long-time photothermal treatment. This could be another reason why the high number of pulses cured electrodes showed poor capacitive performance (discussed later) than two pulses cured electrode.

**Figure 5:**
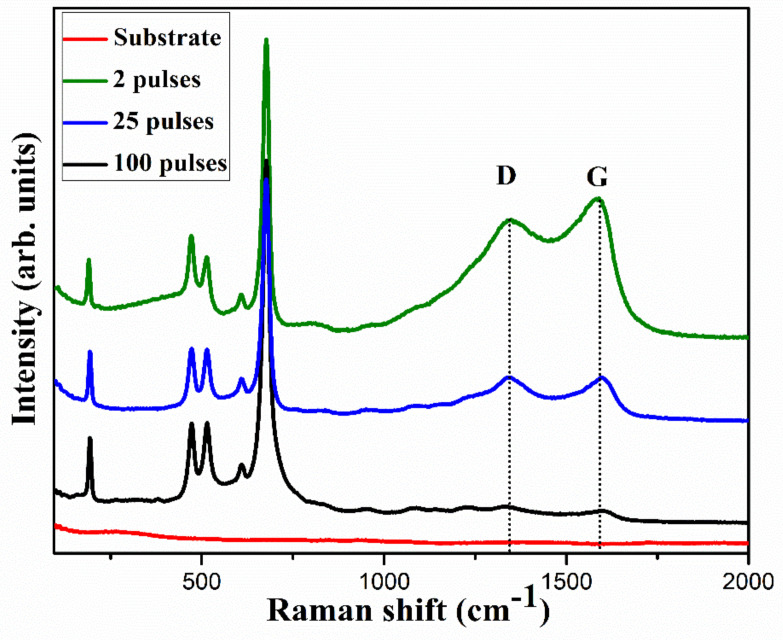
Raman spectra of the as-prepared electrodes, clearly showing the D and G peaks which represent the presence of defect and the graphitic phase of carbon, respectively.

The crystalline structure of the electrodes was analyzed using Raman spectra, as shown in Figure 5. For all the electrodes, the Raman spectra showed peaks associated with vibrational modes of CoO and Co_3_O_4_. The peak centered at 190 cm^−1^ corresponds to F_2g_ mode of CoO [23] while peaks centered at 478, 515, and 610 cm^−1^ correspond to E_g_, F_2g_, and F_2g_ vibration modes of Co_3_O_4_, respectively [11]. The strong peak centered at 680 cm^−1^ corresponds to A_1g_ vibration mode of Co_3_O_4_ [1,11,24]. Additionally, the presence of characteristic D and G peaks, respectively, at 1344 and 1586 cm^−1^ confirm the presence of reduced graphitic oxide in the films [25]. The D and G peaks, respectively, correspond to the disordered carbon and the presence of graphene associated with E_2g_ mode phonons of the sp^2^ graphitic carbon [26]. The presence of the highly conductive graphitic phase of carbon could help improve the performance of the electrode by reducing series resistance during the electrochemical processes [27]. In the Raman spectra, two pulses cured electrode showed strong D and G peaks compared to other electrodes, indicating a higher amount of carbon in the electrode, which agrees with the EDX spectra. The presence of weaker D and G peaks in 25 and 100 pulses cured electrodes could be due to the photothermal evaporation of the carbon component in the electrodes with irradiation of an increased number of pulses. The higher G intensity compared to D intensity is indicative of rich graphitic phase carbon in the two pulses cured electrode.

**Figure 6:**
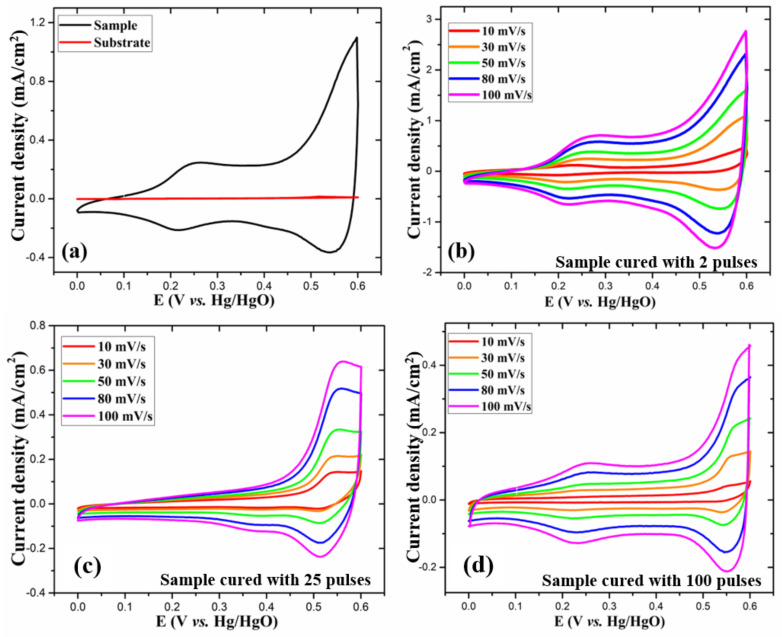
(**a**) CV curves at 30 mV/s scan rate for 2 pulses cured electrode and the bare substrate and (**b**-**d**) CV curves at various scan rates for the 2, 25, and 100 pulses cured electrodes, respectively.

To assess the electrochemical properties of the as-prepared electrodes, electrochemical measurements, namely, cyclic voltammetry (CV), galvanostatic charge–discharge (GCD) and electrochemical impedance spectroscopy (EIS) were performed in 1 M KOH aqueous electrolyte. The measurements were performed in a three-electrode arrangement with as-prepared electrodes, Pt wire and Hg/HgO as working, reference, and counter electrodes, respectively. For all the electrodes, the CV curves were recorded at various scan rates from 10 to 100 mV/s in the potential window of 0–0.6 V. Figure 6a compares CV curves for a bare substrate and the 2-pulses cured electrode at 30 mV/s scan rate. The substrate shows a flat line in the CV curve, confirming its negligible electrochemical contribution. As opposed to rectangular CV curves for EDLCs, the as-prepared electrodes (Figure 6b–d), at all scan rates, showed CV curves with characteristic peaks due to Faradaic redox reactions, indicating the pseudocapacitive behavior of the electrodes. The fact that the CV curves preserved a similar shape even at a high scan rate is indicative of the electrode’s excellent capacitive behavior. It is to be noted that with increasing scan rates, there was a slight shift in the position of the redox peaks due to the increase in internal resistance. The as-obtained CV curves are in agreement with the previously reported studies [4,26,28,29,30] for CoO_x_ electrodes. The presence of the redox peaks in the CV curves is associated with the following reversible chemical reactions of Co_3_O_4_ with electrolyte ions [1,31,32]:

Co_3_O_4_ + OH^−^ + H_2_O = 3CoOOH + e^−^ (1)

CoOOH + OH^−^ = CoO_2_ + H_2_O + e^−^ (2)

To investigate the charge storage characteristics of the as-prepared electrodes, the CV at 30 mV/s scan rate is plotted in Figure 6a. Fascinatingly, at the same scan rate, the two pulses cured electrode shows a significantly larger CV curve area than other electrodes, exhibiting its excellent electrochemical behavior and enhanced charge storage capacity. Furthermore, from the CV curves at various scan rates, the relationship between the measured current (*i*) and scan rate (*v*) can be represented by the following equations [33,34]:

(3) $$i=a\nu^{b}$$

(4) logi=loga+blogv

where *a* and *b* are adjustable parameters.

**Figure 7:**
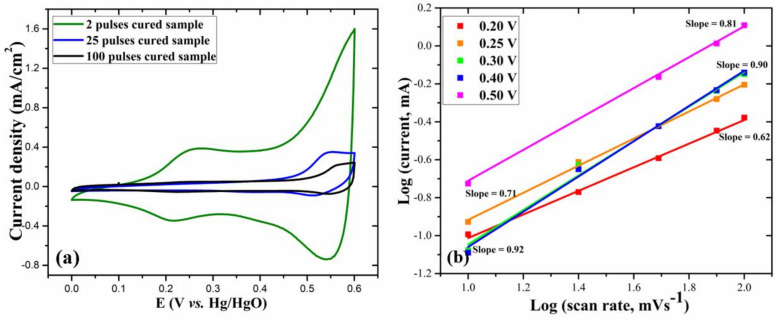
(**a**) CV curves at 30 mV/s scan rate for different electrodes and (**b**) log (*i*) versus log (*v*) plot at various potentials for the 2 pulses cured electrode.

From these equations, with the value of *b*, we can distinguish whether the charge storage mechanism is mainly diffusion-controlled faradaic interactions or double-layer adsorption of ions. If the measured current is proportional to the scan rate (*b* = 1), the electrochemical processes are mainly capacitive, and if the measured current is proportional to the square root of scan rate (*b* = 0.5), the electrochemical processes are mainly diffusion-controlled [35]. From equation (4), for the two pulses cured electrode, we calculated the values of b from the log (*i*) versus log (*v*) plots, as shown in Figure 7b. The b-values at different potentials (V) were in the range of 0.62–0.92, denoting that the charge storage is achieved through the coupled effect of capacitive mechanism and diffusion-controlled Faradaic interactions. As shown in Figure S2 (ESI), for the 25 and 100 pulses cured electrodes, at 0.55 V anodic peak potential, the b-values, respectively, were 0.33 and 0.51, indicating that the charge storage mechanisms are mostly diffusion-controlled.

**Figure 8:**
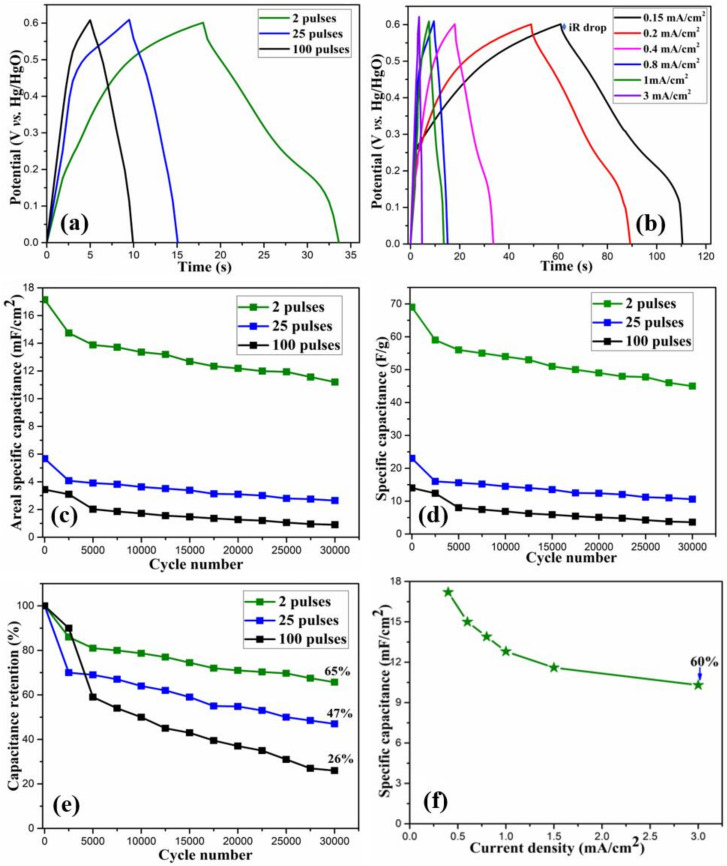
(**a**) Galvanostatic charge-discharge (GCD) curves at 0.40 mA/cm^2^ current density for the samples, (**b**) GCD curves for 2 pulses cured sample, (**c**–**e**) areal specific capacitance, specific capacitance and specific capacitance retention % for all the samples, (**f**) rate capacity measurement for 2 pulses cured sample.

The electrochemical performance of the electrodes was further analyzed by the GCD measurements. As opposed to the triangular GCD curves in EDLCs, the GCD curves for the as-prepared electrodes were non-linear with the presence of sudden drops, also known as internal resistance (IR) drops, at the start of the discharge process. Such non-linear nature of the GCD curves is attributed to the Faradaic redox reactions between the electrode material and the electrolyte, which further confirms the pseudocapacitive charge storage characteristics. Figure 8a shows the GCD curves at 0.4 mA/cm^2^ areal current density for all the samples. It is to be noted that the two pulses cured electrode shows a longer discharge time, indicating its better capacitive behavior. Figure 8b presents the GCD curves at various areal current densities, ranging from 0.15 to 3 mA/cm^2^ for the two pulses cured electrode.

Since the capacitance in pseudocapacitors depends on the certain working potential of the material [36], it is appropriate to calculate the specific capacitance from the GCD curves, instead of CV curves using the following equation [37]:

(5) $$C\;=\;\frac{I\;*\Delta t}{A*\Delta V}$$

where *I* (mA) is the applied current, Δ*t* (s) is the discharge time, *A* (cm^2^) is the area of the electrode, and Δ*V* (V) is the potential window during discharge excluding the IR drop.

From the above equation, the areal specific capacitance at 0.40 mA/cm^2^ constant current density was calculated to be 17.2, 5.6, and 3.4 mF/cm^2^ for 2, 25, and 100 pulses cured electrodes, respectively. Similarly, we also calculated the gravimetric capacitance using Equation (1) and the 2, 25, and 100 pulses cured samples, respectively, showed the initial specific capacitance of 69, 23 and 14 F/g.

The long-term cyclic stability of the electrode is crucial for its practical applications. For the as-prepared electrodes, the cyclic stability was tested by performing continuous GCD measurements at 0.4 mA/cm^2^ constant areal current density for 30,000 cycles. Figure 8c,d, respectively, show areal specific capacitance and specific capacitance after the various number of cycles for all the electrodes. A shown in Figure 8e, among the electrodes, the two pulses cured electrode showed capacitance retention of 65%, indicating its excellent electrochemical stability and ultra-long cycle life. On the other hand, the 25 and 100 pulses cured electrodes showed 47 and 26% capacitance retention after 30,000 cycles, respectively. While most of the previously reported studies [1,2,3,12,28,32,36] on CoO_x_-rGO electrodes have gone up to several thousand (<5000) GCD cycles, we have performed a significantly higher number (30,000) of GCD cycles, and our two pulses cured electrode showed reasonably high capacitance retention, making it a potential candidate for its application in long term devices. Among three electrodes, the specific capacitance for the two pulses cured electrode is comparable to the already reported similar work [38,39]. Additionally, our processing technique is novel, low-cost and ultra-high speed, and the electrodes have excellent cyclic stability, making this work unique and significant in the field of transition metal oxide supercapacitors. For the as-prepared electrodes, the decrease in capacitance after a high number of cycles could be due to the dissolution of the active material, degradation of the electrolyte, change in microstructure due to ions intercalation/deintercalation and active material erosion due to long time exposure to the electrolyte.

Rate capability is a crucial parameter for the electrode’s application. For the two pulses cured electrode, this was performed by carrying out the GCD curves at various areal current densities, as shown in Figure 8f. The electrode showed 60% capacitance retention even at the high applied current density of 3 mA/cm^2^, revealing its excellent rate capacity. With the increase in the applied current, the electrode showed a gradual reduction in specific capacitance. Generally, the specific capacitance is high at low current densities. This is because the electrolyte ions can move not only to the surface but also to the inner pores by penetrating deep into the electrode, taking part in the intercalation and deintercalation process. Due to the high diffusion of the electrolyte ions, effective utilization of the active materials is increased, resulting in high specific capacitance. Contrarily, at high current densities, the movement of ions is limited due to their slow diffusion, which prevents the ions from reaching the inner pores of the electrode. This means that only the surface of the electrode can take part in electrochemical reactions, resulting in the decreased utilization of the active material. Therefore, the specific capacitance is low at high applied currents. The as-prepared two pulses cured electrode showed enhanced electrochemical performance because of: (i) the binder-free growth of the nanostructures on the conductive substrate, (ii) the enhanced structural integrity as porous morphology helps accommodate the volume changes during the charge–discharge processes, (iii) its excellent pseudocapacitive charge storage mechanism achieved through enhanced ion diffusion, and (iv) the higher content of rGO in the electrode which provides structural stability as well as high active surface area for electrochemical reactions.

**Figure 9:**
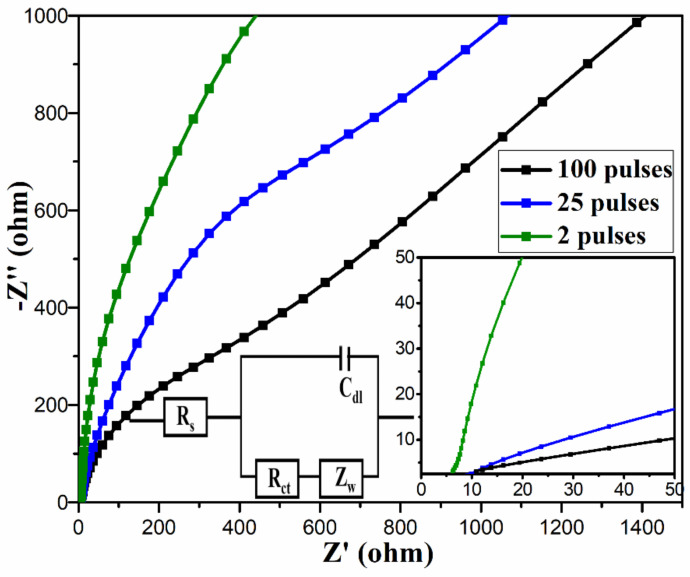
Nyquist plots for all the samples, the insets show a zoom-in of Nyquist plots at high frequency region, and the equivalent circuit.

The electrochemical properties of the electrodes were further studied by electrochemical impedance spectroscopy (EIS) as it is one of the principal methods to analyze the fundamental behavior of the electrodes [40]. The EIS measurements in the form of Nyquist plots were carried out in the frequency range from 100 kHz to 10 mHz with AC perturbation signal of 10 mV shown in Figure 9 with insets showing a zoom-in of Nyquist plot and an equivalent circuit. Generally, the Nyquist plot contains a semicircle in the high-frequency region and a linear section at the low-frequency region. The *x*-axis intercept of the semicircle represents the equivalent series resistance (ESR), also denoted by R_s_, which represents the sum of the resistances of the electrolyte, electrode, and the contact resistance at the electrolyte–electrode interface [41]. The diameter of the semicircle gives charge transfer resistance due to faradaic reactions and electrochemical double-layer capacitance [42,43,44]. A linearly increasing line in the low-frequency region of the Nyquist plot is due to the diffusion of the ions in the electrode, and this could be represented by Warburg resistance (Z_w_) in the equivalent circuit [9,31,45]. From the linear section, the charge storage mechanism can be studied. While a vertical line corresponds to the EDLC behavior of the electrode, a vertically increasing line corresponds to the diffusive behavior of the electrode. The Nyquist plots show lower R_s_ (6.2 Ω) for two pulses cured electrode than that for 25 pulses (9.5 Ω) and 100 pulses (11 Ω) cured electrodes, signifying the improved conductivity of the two pulses cured electrode. Furthermore, it was observed that the as-prepared electrodes do not show any semicircle at high-frequency regions, as shown in zoom-in view of Nyquist plot, indicating their low or negligible faradaic charge transfer resistance and excellent ion transport. At low-frequency region, the plot for two pulses cured electrode shows more vertical line than other electrodes, revealing its better capacitive behavior and lower diffusion resistance.

Overall, from the above characterizations and analysis, we observed that the two pulses cured electrode showed better electrochemical performance than 25 and 100 pulses cured samples, both in terms of specific capacitance and capacitance retention. This could be due to: (i) the desirable highly porous morphology, (ii) its high charge storage capability as shown by significantly larger area for CV curves, (iii) the presence of higher amount of rGO in the sample (as shown by EDX and Raman spectra) as opposed to 25 and 100 pulses cured electrodes since the high number of pulses resulted in reduction in rGO in the electrodes due to thermal evaporation, and (iv) its lower series resistance (6.2 Ω) and better capacitive behavior and lower diffusion resistance as indicated by the almost vertical line at the lower frequency region of the Nyquist plot.

## 4. Conclusions

In summary, we have presented a unique, novel, cost-effective, extremely quick and industrial-scale method to prepare nanostructured metal oxide thin film electrodes in ambient conditions. By using this millisecond processing technique, we have eliminated longer processing times in the case of conventional processing methods. Due to the unique processing mechanism of this technique, the as-prepared electrodes contain porous and robust nanostructures, allowing for faster ion diffusion and enhancing the utilization of the active material during electrochemical processes. The as-prepared two-pulses cured electrode showed initial areal specific capacitance and specific capacitance of 17 mF/cm^2^ and 69 F/g, respectively. Furthermore, even after 30,000 cycles, the electrode showed excellent capacitance retention of 65%, exhibiting its excellent capacitive performance and long cycle life, both of which are crucial requirements for long term applications. We hope this method can be applied to fabricate other metal oxides as well as hybrid electrodes for supercapacitors.

## Data Availability

The data presented in this study are available on request from the corresponding author.

## Supplementary Materials

The following are available online at https://www.mdpi.com/2079-4991/11/3/717/s1, Table S1: Electrode processing parameters, Figure S1: SEM images for the as-prepared 2 pulses cured (a), 25 pulses cured (b) and 100 pulses cured (c) samples, Figure S2: Log (i) versus log (v) plot at 0.55V for 25 and 100 pulses cured sample.
