# Supplementary material for: Preparation of Cobalt Oxide–Reduced Graphitic Oxide Supercapacitor Electrode by Photothermal Processing
Source: Nanomaterials (Basel). 2021 Mar 12;11(3):717. doi: 10.3390/nano11030717 (PMC7999613; doi:10.3390/nano11030717)
Supplement: Supplementary file 1 [file nanomaterials-11-00717-s001.pdf]

Supplementary Materials

Preparation of Cobalt Oxide–Reduced Graphitic Oxide Supercapacitor Electrode by Photothermal Processing

Madhu Gaire *, Najma Khatoon and Douglas Chrisey

Department of Physics and Engineering Physics, School of Science and Engineering, Tulane University, New Orleans, LA 70118, USA; nkhatoon@tulane.edu (N.K.); dchrisey@tulane.edu (D.C.)
* Correspondence: mgaire@tulane.edu

Table S1. Electrode processing parameters.

Applied Voltage (V)	Envelope Width (μs)	μpulses	Fire Rate (Hz)	Pulse Fluence (J/cm^{2})	No. of Pulses
670	1950	8	1.2	7 J/cm^{2}	2, 25, 100

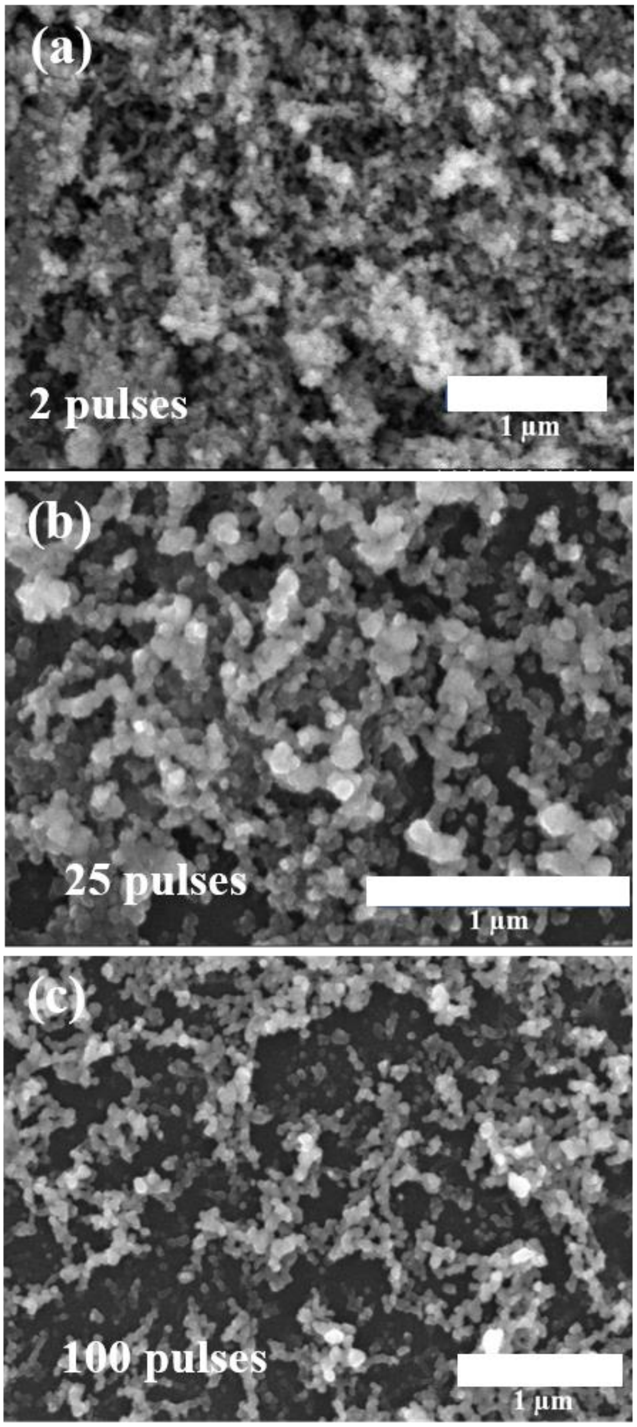

Figure S1. SEM images for the as-prepared 2 pulses cured (**a**), 25 pulses cured (**b**) and 100 pulses cured (**c**) electrodes.

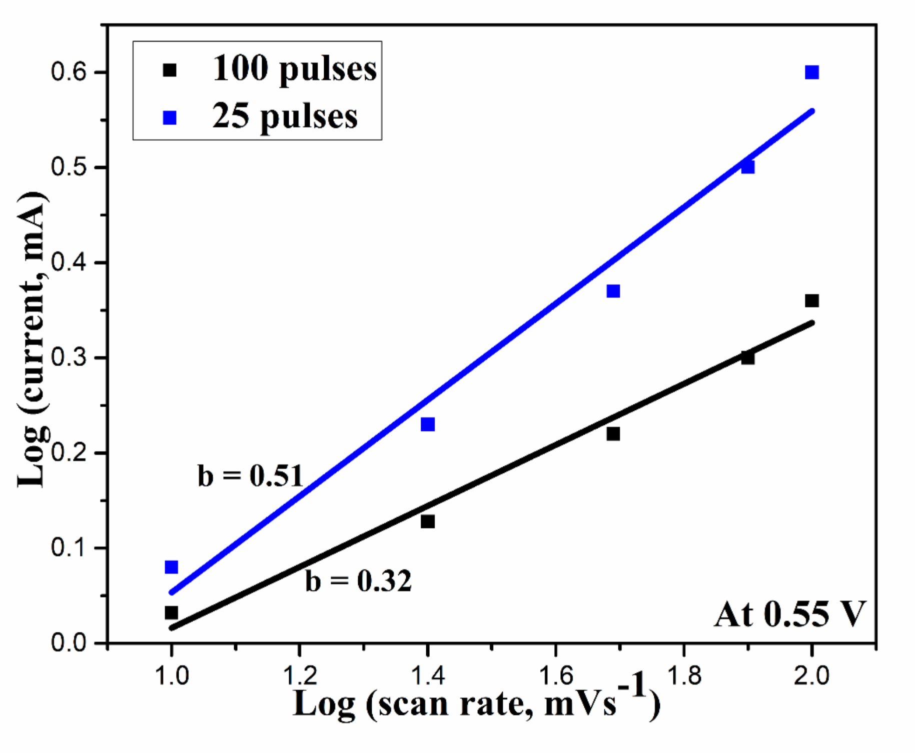

Figure S2. Log (i) versus log (v) plot at 0.55V for 25 and 100 pulses cured electrodes.
